# Evolutionary genomics of endangered Hawaiian tree snails (Achatinellidae: Achatinellinae) for conservation of adaptive capacity

**DOI:** 10.7717/peerj.10993

**Published:** 2021-04-22

**Authors:** Melissa R. Price, Michael G. Hadfield, Ingrid S.S. Knapp, Robert J. Toonen, Zac H. Forsman

**Affiliations:** 1 Department of Natural Resources and Environmental Management, University of Hawaiʻi at Mānoa, Honolulu, HI, USA; 2 Kewalo Marine Laboratory, Pacific Biosciences Research Center, University of Hawai’i at Mānoa, Honolulu, HI, USA; 3 Hawai’i Institute of Marine Biology, University of Hawai’i at Mānoa, Kāne’ohe, HI, USA

**Keywords:** Island species radiation, Colonization, RADseq, Mitochondrial genome, Phylogenomics, Conservation genetics, Translocation, Captive rearing

## Abstract

Phylogenomic studies can provide insights into speciation, adaptation, and extinction, while providing a roadmap for conservation. Hawaiian tree snails are a model system for an adaptive radiation facing an extinction crisis. In the last 5 years, nearly all populations of Hawaiian tree snails across the 30 remaining species in the subfamily Achatinellinae (Achatinellidae) have declined from hundreds or thousands in the wild down to undetectable levels. Nearly 100 species historically occurred across dramatic environmental gradients on five of the Hawaiian Islands, but habitat loss, overcollection, and predation by invasive species have decimated populations. As such, this system offers the opportunity to integrate efforts to conserve evolutionary potential into conservation planning for a rapidly declining subfamily. Here, we used genome-wide, restriction-site associated DNA sequencing (RADseq), along with mitochondrial genome reconstruction, to resolve evolutionary relationships to inform conservation efforts. Phylogenetic analysis of nearly 400k genome-wide SNPs from 59 populations and 25 species across six genera in the family Achatinellidae, was generally concordant with taxonomy, geography, and mtDNA with several notable exceptions; mtDNA was unable to resolve some deeper nodes (e.g., the monophyly of *Achatinella*), while SNP data did not resolve as many shallow nodes. Both phylogenetic and coalescent analysis revealed deep divergences between populations within *Achatinella mustelina* that were consistent with species-level differences. Given cryptic species-level divergence within populations that are geographically proximate, they are at higher risk of extirpation from invasive predators and climate change than previously assumed. This study clarifies evolutionary relationships within this model system for adaptive radiation, forming the basis for conservation strategies such as translocation, captive rearing, and hybridization trials to prevent the loss of capacity to adapt to rapidly changing environmental conditions.

## Introduction

Habitat loss, predation by introduced species, and over-harvesting by collectors led to the extinction of more than 50 species of tree snails in the subfamily Achatinellinae in the last century, resulting in the declaration of all remaining species in the genus *Achatinella* as Endangered (Hadfield & Mountain, 1980; U.S. Fish & Wildlife Service, 1981; Hadfield, 1986). In the last 5 years most of the remaining species have dramatically declined from hundreds or thousands in the wild down to nearly undetectable levels (D. Sischo, 2018, personal communication). Recent declines are largely due the introduction of the Rosy Wolf Snail (*Euglandina rosea*), a carnivorous snail native to Florida that was introduced as a biocontrol measure for the agricultural pest snail *Achatina fulica*, also a non-native species to the islands (Hadfield, Miller & Carwile, 1993; Meyer et al., 2017). The impacts of this invasive predator have been devastating to native land snails in Hawai‘i (Cowie, 1998), perhaps because Rosy Wolf Snails track slime trails to hunt prey (Meyer & Cowie, 2010; Holland et al., 2012), a particularly efficient strategy that allows them to detect land snails of multiple size classes and species.

By reducing mortality from invasive predators such as rats, Jackson’s chameleons, and the Rosy Wolf Snail (Rohrer et al., 2016), predator-free in situ exclosures and captive rearing efforts may allow populations to recover and reach numbers typical of historic populations (Hadfield & Miller, 1989; Price et al., 2015). To date, five exclosures have been constructed in the Wai‘anae Mountains on the island of O‘ahu, Hawai‘i, USA, protecting two species of tree snails (*Achatinella concavospira, A. mustelina*), as well as other native invertebrates and plants. Two other exclosures, one in the Ko‘olau Mountains of O‘ahu, and one on the island of Lāna‘i, protect four additional species within the subfamily (*A. lila, A. sowerbyana, Partulina semicarinata, P. variabilis*). Thus, of approximately 30 remaining species in the subfamily Achatinellinae, only six are protected inside predator-free exclosures. Further, the majority of these protected species are within the genus *Achatinella*, and none of the species in the genera *Newcombia* or *Perdicella* are protected by predator-free exclosures.

Given the recent collapse of populations and the high potential for recovery with the construction of predator-free exclosures, conservation managers wish to combine populations in existing and future exclosures in ways that minimize the potential for outbreeding depression, maximize genetic diversity and future evolutionary potential, and maximize the number of tree snail species protected in the existing exclosures. As tree snails in the subfamily Achatinellinae take approximately 5 years to reach maturity and give live birth to less than 10 offspring per year (Hadfield & Mountain, 1980; Hadfield & Miller, 1989; Hadfield, Miller & Carwile, 1993; Price et al., 2015), lab experiments to test concerns regarding hybridization, outbreeding and inbreeding depression are not feasible in a timely manner. Species in Achatinellinae occur over elevational gradients with varying climatic regimes, so translocation of individuals into existing exclosures must take into account habitat suitability and the potential for outbreeding depression when combining populations. Furthermore, limited mobility and habitat fragmentation have resulted in minimal gene flow among many of the remaining populations for the last 10,000 years (Holland & Hadfield, 2002). Given the need for timely information regarding multi-level taxonomic relationships among subpopulations, populations and species, genetic tools were deemed the most efficient approach to obtain information for translocation decision-making.

Of the endangered tree snails that are extant in the wild, *Achatinella mustelina* (Mighels, 1845) is the most abundant and locally widespread, with at least 2,000 individuals remaining in the wild (Holland & Hadfield, 2002). Studies of *A. mustelina* based on a fragment of a single barcoding gene, cytochrome oxidase I (COI), synonymized many of the subspecies that had been previously characterized based on shell morphology and identified six evolutionarily significant units (ESUs) whose distributions generally correlated with geographic features such as ridgelines (Holland & Hadfield, 2002; Holland & Hadfield, 2007b). A second study examining 23 species across the subfamily Achatinellinae identified an O‘ahu origin of the lineage, and multiple colonization events across the Hawaiian Islands (Holland & Hadfield, 2004). In the last 20 years the field of genetics has transitioned from these types of single marker studies to include a growing number of loci, increasingly incorporating genomic methods (Stapley et al., 2010), but the application of genomic tools to non-model organisms has only recently become possible (Garvin, Saitoh & Gharrett, 2010). Reduced-representation sequencing is increasingly affordable and becoming widely applied to non-model organisms (Helyar et al., 2011; Toonen et al., 2013), providing increased phylogenetic resolution (Rokas et al., 2003), and improving the understanding of species and population-level relationships that single markers are not capable of resolving (Harrison & Kidner, 2011). Furthermore, the use of multiple loci that vary in their rates of mutational accumulation allows examination of relationships at short and long evolutionary timescales (De La Harpe et al., 2017).

The broad goal of this study was to resolve the evolutionary relationships across the subfamily Achatinellinae with a focus on one of the most threatened genera, *Achatinella*, to inform conservation efforts and future work on this model system for conservation and adaptive radiations. In particular, given constraints regarding the number of available predator-free exclosures, we wished to identify relationships so that managers may combine species in exclosures in ways that maximize evolutionary potential, and minimize the likelihood of hybridization between taxa, while considering population-level concerns such as maximizing genetic diversity and minimizing inbreeding and outbreeding depression. We examined whether the relationships previously uncovered utilizing a single barcoding mitochondrial gene, COI (Holland & Hadfield, 2002, 2004, 2007b), were consistent with a reduced representation genome-wide approach. To minimize the risk of injury from tissue sampling we used minute foot biopsies or mucous swabs which required several individuals from each population to be pooled prior to genotyping to yield sufficient DNA for genomic libraries. We followed the ezRAD protocol (Toonen et al., 2013; Knapp et al., 2016), a reduced representation genotyping method designed for non-model organisms without the need for specialized laboratory equipment. An additional benefit of this approach is that it routinely generates large contigs of high copy-number loci, including nearly complete mitochondrial genomes for each library, in addition to tens of thousands of “stacks” of reads suitable for SNP genotyping. We evaluated whether the relationships among populations in the subfamily were consistent with previously identified evolutionary relationships by constructing genome-wide, SNP-based and mitochondrial genome sequence-based phylogenies that included species in all four genera within the subfamily Achatinellinae (*Achatinella, Newcombia, Partulina, Perdicella*), as well as from two outgroup taxa that are genera within the family, but outside of the subfamily Achatinellinae (*Auriculella*, *Tornatellides*). In addition, we conducted a coalescent-based, species-tree approach to examine genetic structure within the most abundant species, *Achatinella mustelina*.

## Methods

### Field Sites

**Figure 1:**
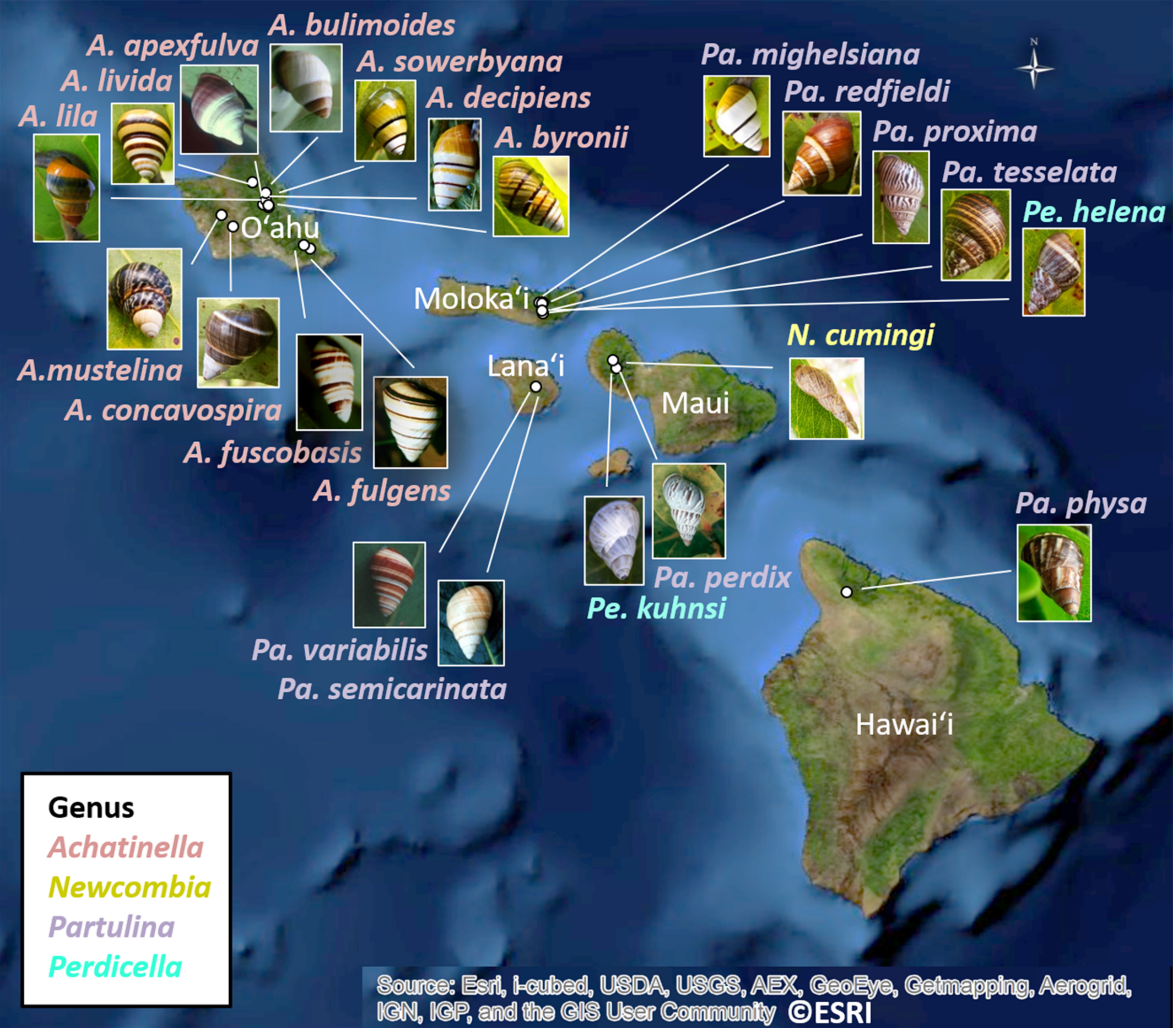
Map showing locations of 22 species in the subfamily Achatinellinae across the Hawaiian Archipelago (outgroup species in the study are not pictured). The name of each species is color-coded by genus. All species have a range of a single-island and a single-mountain range. Many species are only found in a single valley. Source: ©ESRI.

**Figure 2:**
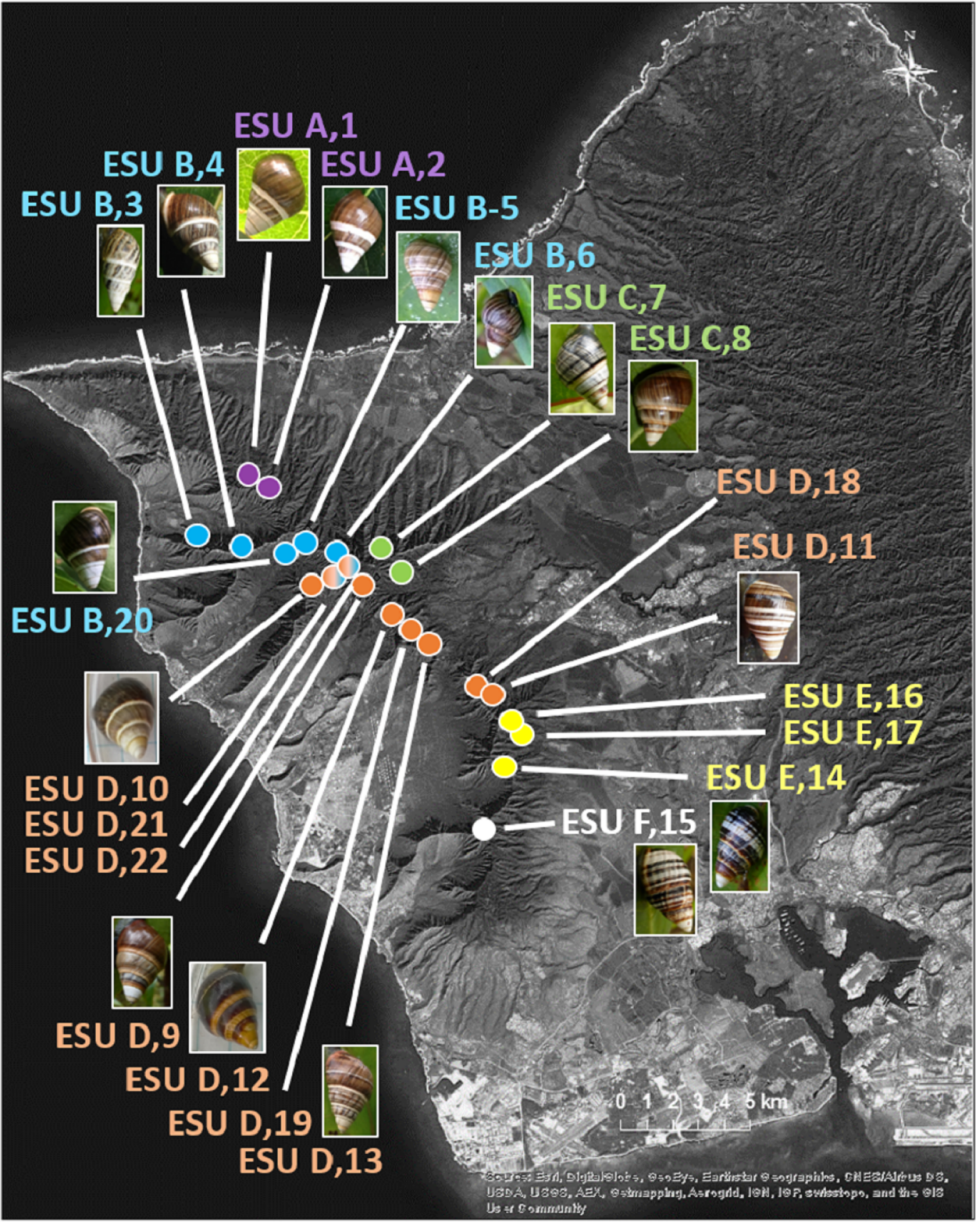
Map of populations of the species *Achatinella mustelina* in the Wai‘anae Mountain Range on the island of O‘ahu. Population points are color-coded by evolutionary significant unit (ESU).

The 25 species in this study (Fig. 1, three outgroup species not pictured) are single-mountain range and single-island endemics, but most remain over only a 1–10 km range, or in a captive rearing facility, due to heavy predation by invasive species in recent decades (Hadfield, 1986). Species in the genus *Achatinella* occur entirely on the island of O‘ahu (Fig. 1). Species in the genera *Partulina* and *Perdicella* occur almost entirely within Maui Nui, where three islands with tree snails, Lāna‘i, Moloka‘i, and Maui, were intermittently connected by land bridges as sea levels rose and fell during the Pleistocene, influencing connectivity and dispersal (Fig. 1; Holland & Hadfield, 2007a; Jones & Jordan, 2015). Species in the genera *Newcombia* and *Perdicella* historically occurred on Moloka‘i and Maui, and species in the genus *Partulina* historically occurred on Lāna‘i, Moloka‘i, Maui, O’ahu and Hawai‘i (Fig. 1). The range of *Achatinella mustelina*, currently the most abundant species in the subfamily, extends about 25 kilometers north to south in the Wai‘anae Mountain Range on the island of O‘ahu along elevational clines of 400–1,200 m (Fig. 2). These elevational clines correlate with rainfall and temperature, with a rain shadow effect between the windward and leeward sides of the mountain range.

### Sample collection and DNA extraction

We conducted non-lethal sampling protocols under US Fish and Wildlife permit TE-826600-14 with limited sampling of rare populations. Samples from 59 populations among 25 species and six genera were obtained from wild or captive-reared populations, or from deceased individuals from captive-reared populations preserved in 95% ethanol. Between November 2012 and June 2016 small tissue biopsies were collected in a nonlethal manner from 1–50 living individuals per wild or captive population and individually preserved in 100% ethanol until DNA extraction, following methods similar to Thacker & Hadfield (2000) in which approximately 1–2 mm^3^ of tissue were removed by a sterile razor blade from the foot of the snail. All accessible populations where it was expected that a minimum of 15 individuals could be found were sampled. We sampled between 1–50 individuals per population (mean ± standard deviation = 22 ± 17) to attempt to capture genetic diversity in the population (Table S1). In some cases very few individuals could be found, requiring additional precautions; for example for *Achatinella concavospira*, mucus from individuals was collected by gently swiping a sterile polyester-tipped swab along the foot of the snail, then placing the tip in a dry tube at −20 °C until extraction. Mucus was collected, rather than tissue, to minimize potential risk in a species with few individuals remaining. DNA was individually extracted from tissue or swab samples using a DNeasy Blood and Tissue Kit (Qiagen, Hilden, Germany) according to the manufacturer’s protocol. Extracted DNA was quantified using the Biotium AccuClear Ultra High Sensitivity dsDNA quantitation kit with seven standards. Equal quantities of DNA from each individual within a population were pooled to a total of 1 µg. From these pools, libraries were prepared for genome sequencing using the ezRAD protocol (Toonen et al., 2013) version 2.0 (Knapp et al., 2016). Samples were digested with the frequent cutter restriction enzyme DpnII from New England Biolabs^®^. They were then prepared for sequencing on the Illumina^®^ MiSeq using the Kapa Biosystems Hyper Prep kit following the manufacturer guidelines with the exception of the size selection, which targeted DNA fragments between 350–700 bp for this application. All samples were amplified after size selection for the recommended cycles to generate 1 µg of adapter-ligated DNA. Once complete, all libraries were run on a bioanalyzer and with qPCR to validate and quantify them to ensure equal pooling on the MiSeq flow cell. Quality control checks and sequencing were performed by the Hawaiʻi Institute of Marine Biology Genetics Core Facility.

## Analyses

### Mitochondrial Genomes

Complete or nearly complete mitochondrial genomes were extracted from each library using a method described previously (Price et al., 2016a, 2016b, 2018), briefly; raw Illumina reads were sorted by barcodes and imported into Geneious v.6.1.8 (Biomatters Ltd., Auckland, New Zealand). Forward and reverse reads were grouped into a paired list and quality trimmed to allow no more than a 0.1% chance of error and adaptor trimmed from both ends of reads allowing no mismatch and a minimum overlap of 8 bp. Each library was then mapped to the whole mitochondrial reference mitogenomes of *Achatinella fulgens, A. mustelina*, *A. sowerbyana, Partulina redfieldi*, or *Perdicella helena* (Price et al., 2016a, 2016b, 2018), whichever resulted in the highest number of mapped sequences. Through an iterative process described below, whole or partial mitogenomes for all populations were constructed. Reads were initially mapped to published mitogenomes. The alignments of mapped sequences were visually inspected, and a consensus sequence was generated. This consensus sequence was then used as a reference for the next iteration, in which all sequences produced for a given species or population were mapped against the consensus sequence achieved in the previous round of alignment. This process was repeated until either the complete mitochondrial genome was obtained or until successive iterations did not result in additional mapped sequences compared to the previous round. In total, 969–7,474 reads per population mapped to the reference mitochondrial genome, with mean library depth of coverage of 46.3X ± 73.6X (mean ± standard deviation). The mitochondrial genomes were composed of 13 protein-coding genes, two rRNA genes, and 22 tRNA genes, similar to other Panpulmonata (White et al., 2011). Next, multiple sequence alignments of all complete or partially complete mitochondrial genomes were performed with MUSCLE v.3.8.31 (Edgar, 2004) under default parameters. Visual inspection of the alignment found no regions that appeared to be poorly aligned, although there were several regions with indels corresponding to non-protein coding regions. Jmodeltest v.2.1.10 (Darriba et al., 2012) was used to select the best fitting model (GTR+I+G) using the AIC criteria. Maximum likelihood trees were generated with RAxML v. 8.1.16 (Stamatakis, 2014) with the GTR+I+G model and optimization of rate parameters and bootstrap support values based on 1,000 replicates on the “raw” alignment and an alignment with columns with >60% gaps stripped. The alignment was also run with PhyML with SMS model selection (Lefort, Longueville & Gascuel, 2017) implemented in PhyML v.3.0 (Guindon et al., 2010), which also selected the GTR+I+G model and run for 1,000 bootstrap replicates, using the Montpellier Bioinformatics Platform (http://www.atgc-montpellier.fr/). In addition, we examined the potential effects of modeling the partitions and codon positions of the mitochondrial genome using the program IQ-TREE v.1.6.12 (Minh et al., 2020). We used annotations from NC_030190.1 *Achatinella mustelina* to create an annotation table, which was uploaded using the partition model function (Chernomor, Von Haeseler & Minh, 2016) via the IQ-TREE web server (Trifinopoulos et al., 2016). The sequence alignments and trees are available on the following GitHub repository: https://github.com/zacforsman/snaildata.

### Genome-wide SNP analyses

To process raw sequencing reads, we used the dDocent v. 2.2.19 (Puritz, Hollenbeck & Gold, 2014) pipeline, which is a bash wrapper script that includes the program Cutadapt (Martin, 2011) for read trimming, BWA-MEM (Li, 2013) for read mapping, and FreeBayes (Garrison & Marth, 2012) for SNP calling, We focused in first on loci shared across the genus *Achatinella*, since this genus is a major focus of conservation efforts and is listed as endangered, and *de-novo* assembly of all of the loci shared across all libraries was not computationally tractable within a reasonable time frame (e.g., weeks) with our available resources. *De-novo* assembly was first performed on members of the *Achatinella* genus (*n* = 39) to generate a reference sequence that all samples were subsequently mapped to. The *de-novo* assembly options were: Clustering_Similarity% = 0.90, Mapping_Reads = Yes; Mapping_Match_Value = 1; Mapping_MisMatch_Value = 4; Mapping_GapOpen_Penalty = 6. All libraries were then mapped to the *Achatinella* reference produced by dDocent, using default mapping, except the program Freebayes v.1.0.2-29 (Garrison & Marth, 2012) was used to call variants from the merged bam file produced by the dDocent pipeline, with “stringent filters” enabled, ignoring multi-nucleotide polymorphisms and complex events, under the pooled continuous model (i.e., -0 -z .1 -u -K --min-repeat-entropy 1 -V) in order to account for the pooled nature of the samples. The assemblies were visually inspected using Tablet v.1.13 (https://ics.hutton.ac.uk/tablet/) and visual inspection of several dozen loci indicated the assemblies appeared clean with clear rows of SNPs without obvious assembly artifacts such as poor overlap or poor clustering between read groups.

The vcf files were imported into TASSEL v.5 (Bradbury et al., 2007) for a preliminary PCA analysis. The PCA plots provided rapid visualization of the effects of a broad range of filtering parameters (such as missingness, depth, and allele frequency) on clustering of major groups providing a rapid and relatively assumption free method of determining the sensitivity of major clusters to filtering settings, guiding selection of parameters for further exploration using phylogenetic tools. VCFtools v.0.1.15 (Danecek et al., 2011) was used to determine depth and heterozygosity for each library and to filter three datasets for phylogenetic analysis; the “minimally filtered” dataset that retained SNPs with a minimum quality score of 30, depth of 3×, with no limit on missing data per locus, resulting in 391,283 SNPs; the “medium filtered” dataset retained SNPs with a minimum quality score of 30, depth of 5×, and a maximum of 5 missing taxa per locus resulting in 16,255 SNPs; and the “strict filtered” dataset retained SNPs with a minimum quality score of 40, depth of 10×, and a maximum of 10 missing taxa per locus resulting in 14,674 SNPs.

For phylogenetic analysis, the vcf files were converted to fasta format using the program VCFkit (Anderson lab 2014) using the phylo fasta command. This generated a consensus for each SNP locus, reducing ambiguities in the resulting fasta file. Although allelic information was lost using this consensus approach, the most common allele at each site may be more likely to be informative for pooled samples with a relatively high proportion of fixed differences. Jmodeltest v.2.1.10 (Darriba et al., 2012), was used to determine the best fitting model for each dataset (GTR+G regardless of filtering settings) using the AIC criteria. We generated maximum likelihood trees using RAxML v.8.1.16 (Stamatakis, 2014) with the GTRGAMMA model, ascertainment bias correction, and optimization of rate parameters and bootstrap support values based on 500 replicates.

### Estimation of species trees from SNP data

We used the SNAPP (Bryant et al., 2012) package within the BEAST2 (Bouckaert et al., 2014) platform to estimate species trees from SNP data, focusing on the relationships among the ESUs (Evolutionarily Significant Units) of *A. mustelina* which were delineated in a previous study utilizing a single mitochondrial gene (Holland & Hadfield, 2002). SNAPP uses coalescent modeling to infer species trees directly from biallelic markers (Bryant et al., 2012). SNAPP parameters were estimated following guidance from Leaché & Bouckaert (2018); briefly, SNP data was converted directly from the “Final.recode.VCF” file produced by the dDocent pipeline (this file consists of SNPs present in at least 90% of the samples, therefore minimizing missing data), filtered with VCFtools to contain one SNP per contig, and converted into binary SNP format using PGDspider v.2.1.1.5 (Lischer & Excoffier, 2012). ESU populations were defined a priori, mutation rates were directly estimated, non-polymorphic sites were excluded, priors were at default values, and the coalescent rate was estimated during the MCMC run, which was run with a chain length of 1,000,000 and a burnin of 10,000. The program Tracer v.1.7.1 (Rambaut & Drummond, 2016) was used to examine change in likelihood scores and the ESS (Effective Sample Size) scores to determine if the run had reached convergence. DensiTree v.2.2.7 (Bouckaert & Heled, 2014) was used to examine tree topologies, and TreeAnnotater v.2.6.2 (Rambaut & Drummond, 2017) was used to calculate the maximum clade credibility tree, which was visualized in FigTree (Rambaut, 2014).

## Results

After cleaning and pairing forward and reverse reads, we obtained a total of 301,350,630 sequences from between one and 22 populations each from 25 species across six genera, including three species from two subfamilies in the family Achatinellidae (Auriculellinae, Tornatellididae; Table S1).

### Mitochondrial genomes

**Figure 3:**
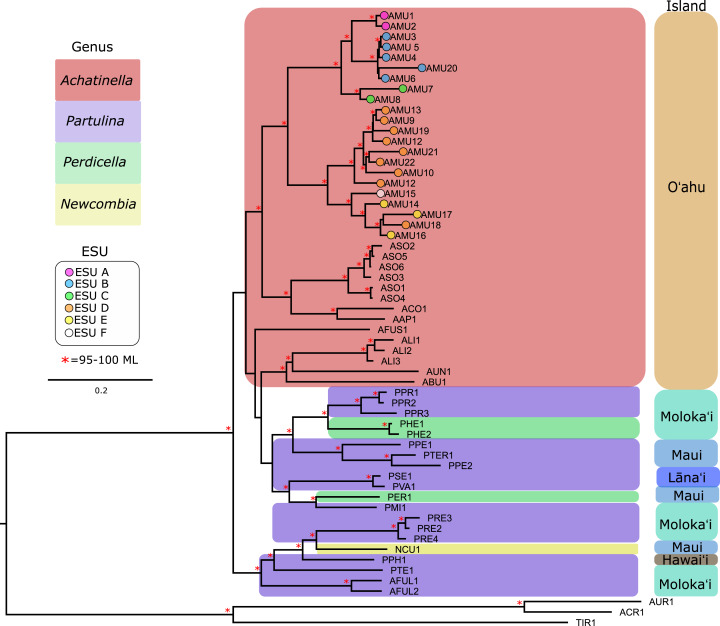
Mitochondrial genome Maximum Likelihood tree. Complete and nearly complete mitochondrial genomes from populations of *Achatinella mustelina* and outgroup taxa were used to construct the tree. The resulting tree was consistent with relationships identified using a single-gene approach in a previous study (Holland & Hadfield, 2002) that originally delineated the ESUs (Evolutionarily Significant Units). Only ML (Maximum Likelihood) support values above 95% are shown as red asterisks.

Our approach captured most of the mitochondrial genome (mean ± SD = 14,098 ± 2,473 bp, or 69–100%; range = 6,041–16,915 bp, or 35–100%) for the majority of the populations that were examined (Table S1). Previously published mitogenomes range in length from 15,187–16,793 bp (Price et al., 2016a, 2016b, 2018). The final multiple sequence alignment was 23,992 bp long, with 39.7% of the alignment consisting of gapped sequences from missing data. To examine the effects of gaps on the mitochondrial genome phylogeny, alignment columns that consisted of more than 60% gaps were removed, resulting in a 14,638 bp alignment with 10.9% gapped positions. These trees yielded highly similar results, although the gap-stripped alignment had overall higher bootstrap support and consistent branch lengths and is presented here (Fig. 3). The partition modeled tree generated with IQ-TREE, and with PhyML also yielded highly similar results (see https://github.com/zacforsman/snaildata for newick files), indicating that the results were robust to model selection.

The mitochondrial genome tree revealed three major clusters across the subfamily, that correspond to the same three major clades identified by Holland & Hadfield (2004) based on a single mitochondrial gene (COI). These three clusters indicate that *Achatinella, Partulina*, and *Perdicella* are polyphyletic (although not strongly supported by bootstrap pseudoreplicates) (Fig. 3). The mitochondrial genome tree was nearly completely resolved with high statistical support for most nodes across the tree, with the interesting exception of lack of support for the deep polyphyletic taxa. Within *Achatinella mustelina*, the 22 populations generally grouped into clusters consistent with ESUs identified in a previously published study (ESUs; Fig. 3). Further, the clustering of ESUs formed two deeply divergent major groups (ABC, DEF), with a level of divergence similar to species-level differences among other species in the subfamily (Fig. 3).

### Genome-wide consensus SNP approach

**Figure 4:**
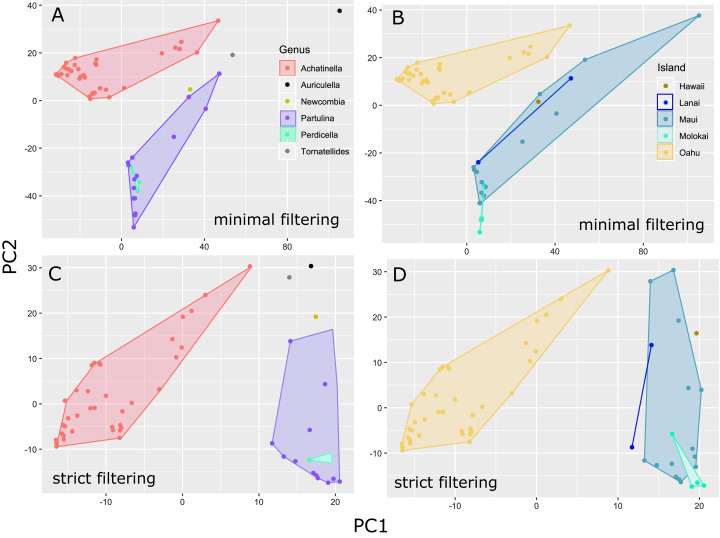
Principle Components Plot showing genetic structure for contrasting SNP filtering settings. The “minimally filtered” dataset consisted of 391,283 SNPs , while the “strict filtered” dataset consisted of 113,128 SNPs. (A) Minimal filtering shaded by genus; (B) minimal filtering shaded by island; (C) strict filtering shaded by genus; (D) strict filtering shaded by island.

**Figure 5:**
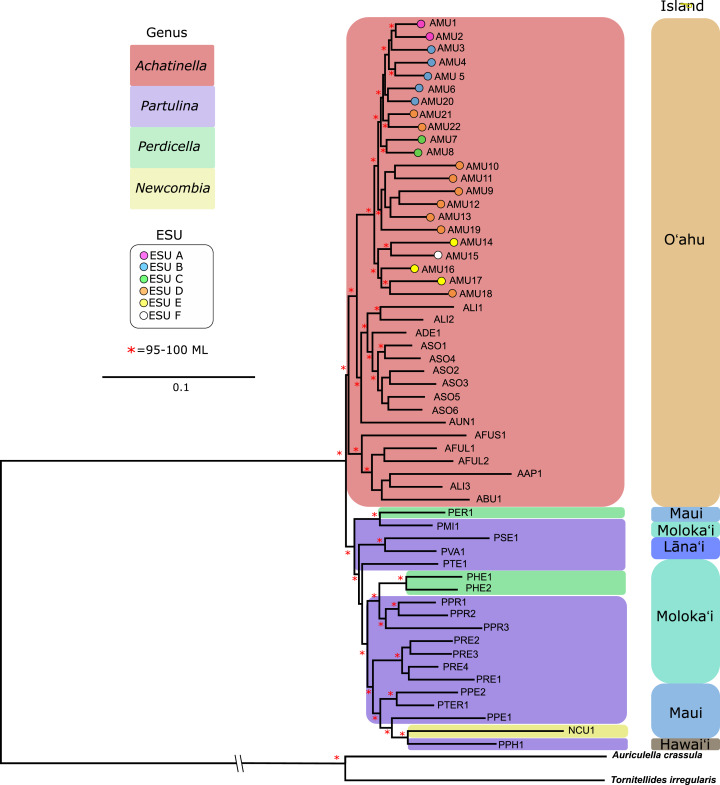
Maximum likelihood tree of the “minimally filtered” genome-wide single nucleotide polymorphisms (SNPs) dataset consisting of 391,283 SNPs. The genome wide SNP tree resolved Achatinella as monophyletic with a strongly supported clade distinct from the other three genera.

The general groupings and evolutionary relationships were robust to a range of filtering settings according to PCA plots: regardless of filtering settings, clusters consistently formed by island biogeography and genus, although the “minimally filtered” dataset resulted in higher resolution and tighter clustering within the major groups (Fig. 4). Similarly, RAxML trees of each data subset revealed that increased filtering resulted in less resolution and lower statistical support, with the “minimally filtered” dataset resolving reciprocal monophyly between *Achatinella* and other genera (Fig. S1). The “minimally filtered” dataset included 391,283 SNPs. This dataset resolved species in the subfamily into two major clades (Fig. 5). In contrast to previous studies that relied on only a single mitochondrial marker, and our mitochondrial genome tree, the genome wide SNP tree resolved *Achatinella* as monophyletic with a strongly supported clade distinct from the other three genera. Species in the genera *Newcombia, Partulina* and *Perdicella* fell out into a second clade comprised of species largely on the historically connected islands of Maui Nui (Molokaʻi, Lānaʻi, Maui), and a single species on the island of Hawai‘i, the youngest island. The “minimally filtered” SNP tree was well resolved with high statistical support for most nodes across the tree and was generally more concordant with taxonomy and geography than the mitochondrial genome tree, although there were fewer taxa that clustered according to ESUs defined by mitochondrial markers for populations of *A. mustelina*.

### Coalescent analysis from SNP data

**Figure 6:**
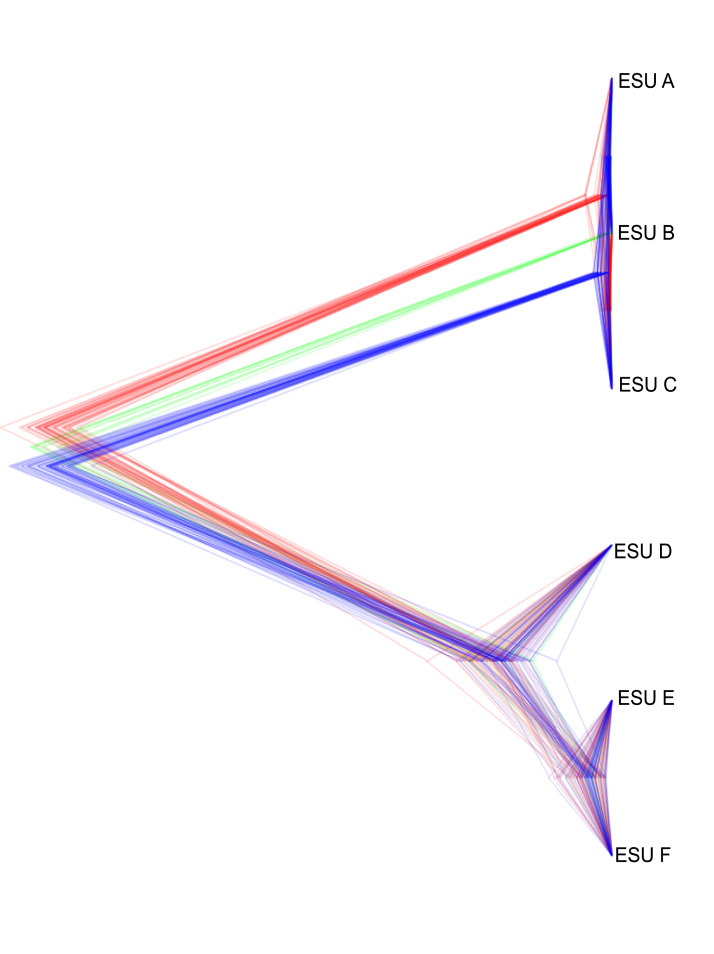
Summary of the posterior distribution of species trees and the topology with the best support. Densitree diagram of the coalescent analysis; blue lines represent the most frequent topology while red and green indicate less frequent alternatives.

The relationships between ESUs, as determined by coalescent analysis of binary SNP data, was consistent with the mitochondrial genome sequence based phylogenetic analysis, and with the genome-wide SNP approach. ESUs A, B, and C formed a closely related cluster and ESUs D,E, and F formed another (Fig. 6). ESUs A, B, and C had shallow divergence, with topological variation that could be due to introgression or sharing of ancestral polymorphism, while ESUs E and F were more closely related to each other than to ESU D, with minimal variation between species trees (Fig. 6). The formation of clades for ESUs A, B, and C as well as for ESUs E and F are very recent and may be ongoing relative to the formation of the other clades.

## Discussion

In this study, an unprecedented volume of data from careful non-lethal sampling provided new insights into the formation and expansion of the Achatinellinae across the Hawaiian Archipelago, while highlighting specific areas for focusing conservation efforts and preventing the loss of adaptive capacity. With reconstructed mitochondrial genomes, combined with a genome-wide SNP approach, we were able to sample most of the remaining extant populations and species throughout the subfamily, providing a roadmap for future work on this model system for adaptive radiation and conservation.

Although the primary goal of this study was to resolve phylogenomic relationships to guide conservation, this dataset is highly geographically structured and may provide fertile ground for a more detailed examination of phylogenetic and population genetic analysis. The non-lethal sampling methods, including swab sampling of mucus, successfully resulted in an unprecedented amount of data from these at-risk populations; however, it was still necessary to pool samples to produce sufficient quantity and quality of DNA for genomic methods. We used a consensus-based SNP phylogenomic approach to accommodate the pooled nature of the data, and to focus on loci that approach fixation as may be expected for more distantly related species, with a coalescent approach to examine recent divergence of key groups of interest in more detail.

The data revealed that populations of *Achatinella mustelina* are likely in the process of undergoing speciation, with cryptic divergence among populations at levels comparable to, or deeper than, accepted species within the family, based on both mitochondrial and genome wide SNP phylogenetic and coalescent analyses. Previous studies based on single mitochondrial markers have identified ESUs A, B, and C on a well-defined, separate branch from ESUs D, E, and F. The level of genetic differentiation observed from these two groups is consistent with species-level divergence across the subfamily, and relatively deep divergence between the ESUs ABC, and DEF was further supported by coalescent analysis. Although there are no obvious geographic barriers between these groups today, the location of the divide between ABC and DEF is concordant with a geological fault line near the top of Mt. Ka‘ala, which may have formed geographic barriers to gene flow in the past (Presley, Sinton & Pringle, 1997; Sherrod et al., 2007). Further work on shell morphology and genitalia are needed to evaluate whether taxonomic revisions are required, as suggested by these major genetic differences.

In contrast to relationships identified using mitochondrial markers, both the mitochondrial genome in this study and the single mitochondrial marker in previous studies (Holland & Hadfield, 2004), the genome-wide SNP analysis resolved the monophyly of *Achatinella*. Although the reconstructed mitochondrial genomes suggested polyphyly, these deeper nodes were not well supported, and may have resulted from rapid diversification, or past introgression. There was also minor discordance between mitochondrial and nuclear data for several of the shallower nodes, with the mitochondrial data providing higher support for the shallower nodes. Despite the major differences between the types of data (SNP vs mitochondrial genome sequences), how they evolve, and how these contrasting data types fit the assumptions of phylogenetic models (Leaché et al., 2015), there were general similarities between mitochondrial and SNP based phylogenies other than minor differences in resolution between several deep and shallow nodes. The mitochondrial polyphyly of *Achatinella* was robust to several alternative modeling approaches (e.g., unpartitioned model testing with Jmodeltest and SMS model testing and fully partitioned genome models in IQ-TREE), therefore alternative explanations for mitochondrial-nuclear discordance seem more likely. Discordance between mitochondrial and nuclear gene trees is as high as 23% across animal species and may be due to a variety of causes, such as rapid evolution, mutational saturation, recombination, hybridization, and contrasting rates of coalescence (reviewed by Funk & Omland, 2003).

It is worth noting that in this study both the mitochondrial and genome-wide SNP approaches supported polyphyly in the genera *Partulina* and *Perdicella*. *Partulina proxima* and *Perdicella helena*, both species found on Molokaʻi, grouped together on a separate branch from *Perdicella kuhnsi*, found on Maui, and *Partulina variabilis* and *Partulina semicarinata*, species endemic to Lanaʻi. As species in the genus *Perdicella* differ from those of *Partulina* largely on the basis of size, and species in the genus *Perdicella* occur on separate branches, this suggests that dwarfism may have evolved multiple times across the species complex. This study included only two species in the genus *Perdicella* and one species in the genus *Newcombia*, a genus with species that have elongated and narrowed shells relative to the other genera, but future studies should include additional species and populations in the genera *Newcombia*, *Partulina*, and *Perdicella* to examine phylogenetic relationships among species, particularly as they relate to shell shape and size.

Early naturalists attributed speciation in Achatinellinae solely to genetic drift, noting that this subfamily was “still a youthful group in the full flower of their evolution” (Pilsbry & Cooke, 1912). The rapid and/or recent nature of this radiation prevented the clear identification of progression across islands from oldest (O‘ahu in this study at 3.0–2.6 Ma; Clague, 1996) to youngest (Hawai‘i in this study at 0.5–0 Ma; Clague, 1996), as has been identified for a number of other species (Cowie & Holland, 2008; Carlile & Sherwood, 2013; Shaw & Gillespie, 2016; Swenson et al., 2019). Whichever the island of origin for this subfamily (O‘ahu or Maui Nui (2.2–1.2 Ma; Clague, 1996)), diversification was rapid as might be expected from the introduction into novel niche space following colonization of a nearby island (Prentis et al., 2008; Gascuel et al., 2016). Following colonization, the Achatinellinae subfamily evolved over complex geographical space and dramatic precipitation and temperature gradients, and thus adaptation may have been quite rapid as species expanded to fill unexploited niches along environmental gradients early in this subfamily’s history. Within Maui Nui, as sea levels rose and fell throughout the glacial periods of the Pleistocene, land bridges were alternately submerged and exposed between islands, driving reproductive isolation and expansion (Mendelson, Siegel & Shaw, 2004; Gillespie, 2016; Norder et al., 2019), and potentially leading to the higher diversity in this island complex compared with O‘ahu. As such, species in the subfamily Achatinellinae provide an excellent system for examining evolutionary processes in future studies. In particular, due to the genetic structuring over dramatic environmental gradients across multiple mountain ranges and islands, this study system appears ideal for examining the ability to predict adaptation to future conditions using outlier loci.

## Conservation implications

Unfortunately, species in this subfamily have dramatically declined in the last few decades (Hadfield & Saufler, 2009; Holland, Montgomery & Costello, 2010), and in the last 5 years in particular (D. Sischo, 2018, personal communication), such that very few remain at detectable levels in the wild. Our results have important implications for the application of the two management tools currently in use to stave off extinction—*ex situ* captive propagation and predator-free exclosures.

Our results may be used to determine appropriate candidates for hybridization trials, if needed. On January 1, 2019 the last known individual in the species *Achatinella apexfulva*, nicknamed “Lonely George” after the last known Galapagos Tortoise of the same moniker, passed away in a captive rearing facility (D. Sischo, 2019, personal communication). Plans had been underway to potentially hybridize this last known individual with *Achatinella concavospira* (D. Sischo, 2018, personal communication), which mitochondrial studies showed to have only a 2.5% genetic difference. Several species in the captive rearing facility are at similarly low numbers, extirpated from the wild, and at high risk of extinction. Conservation-hybridization may offer one solution to retain the genetic diversity represented in these last remaining individuals, and our study supports this effort by identifying the nearest relatives. Further, these results provide a useful starting point for future studies aiming to delimit species boundaries and identify populations most at risk of extinction.

Currently, five predator-free exclosures exist across the Wai‘anae mountain range, and an additional exclosure is under construction, providing protection for *Achatinella mustelina* and *Achatinella concavospira*. Due to the geographic structuring of *Achatinella mustelina* and the distribution of the predator-free exclosures, currently there are representatives from ESUs A, C, D, E, and F in exclosures. The new exclosure will soon hold populations from ESU B. As droughts become more frequent due to climate change, which is expected to result in increased juvenile mortality (Hadfield, Miller & Carwile, 1993), the predator-free exclosures in the southern range may no longer be suitable for sustaining populations. If ESUs ABC and DEF are in fact separate species for which reproductive barriers exist, it may be appropriate to include populations from ESUs A, B, or C in exclosures with populations from ESUs D, E, or F. Captive rearing experiments may assist in elucidating whether this is a viable option and the evolutionary relationships resolved by genomic data from this study may serve as a guide.

Finally, given the divergence within species among populations that are geographically proximate (sometimes within one kilometer), our results indicate that with each population that becomes extirpated by invasive predators, a significant amount of within-species diversity is lost. Previous studies have shown considerable inbreeding within populations (Price & Hadfield, 2014; Price et al., 2015), and for many species less than three known populations remain, so any additional loss raises considerable concern for long-term species viability. We draw attention to concerns that only a handful of the remaining species are currently protected in predator-free exclosures, and highlight that, given the trajectory of extirpation of populations, few (if any) are likely to remain outside of exclosures within the next decade. Construction of new predator-free exclosures on multiple islands, in locations predicted to be suitable both today and under future climatic conditions, are crucial to maintaining species diversity and species persistence into the next century.

The maintenance of evolutionary potential, through thoughtful application of subfamily-wide phylogenomic studies such as this, is crucial to conserving biodiversity. Our study will provide guidance for managers wishing to translocate populations into existing and future predator-free exclosures, to minimize inbreeding and outbreeding risk, maximize genetic diversity, and maintain evolutionary and adaptive potential within the subfamily Achatinellinae. This study system may also provide key insights into speciation, adaptive radiations, and extinction.

## Supplemental Information

10.7717/peerj.10993/supp-1 Supplemental Information 1 Maximum likelihood (ML) trees illustrating how resolution and branch support changed under various SNP filtering settings. **(A)** ML tree of the ‘minimally filtered’ dataset that retained SNPs with a minimum quality score of 30, depth of 3x, with no limit on missing data per locus, resulting in 391,283 SNPs; **(B)** ML tree of the ‘medium filtered’ dataset retained SNPs with a minimum quality score of 30, depth of 5x, and a maximum of 5 missing taxa per locus resulting in 16,255 SNPs; **(C)** ML tree of the ‘strict filtered’ dataset retained SNPs with a minimum quality score of 40, depth of 10x, and a maximum of 10 missing taxa per locus resulting in 14,674 SNPs . Branches with ML (Maximum Likelihood) bootstrap support values above 90% are colored green, while values below ~77% are red, Achatinellidae are highlighted in red, while other genera are in blue font. The hash marks on the outgroup branches indicate that these branches are not to scale.

10.7717/peerj.10993/supp-2 Supplemental Information 2 Tree snail sample attributes. Populations that were sequenced were from the family Achatinellidae, and included species from the subfamilies Achatinellinae, Auricullelinae, and Tornatellidinae. The focal species of this study, *Achatinella mustelina*, included 22 populations from six Evolutionary Significant Units (ESU), named A through F, which were identified in a previous study using the mitochondrial gene COI.

## Additional Information and Declarations

### Field Study Permissions

The following information was supplied relating to field study approvals (i.e., approving body and any reference numbers): Samples were collected under US Fish and Wildlife permit TE-826600-14.

### Data Availability

The following information was supplied regarding data availability: Data is available at NCBI: PRJNA595344: SAMN13548455, SAMN13548456, SAMN13548457, SAMN13548458, SAMN13548459, SAMN13548460, SAMN13548461, SAMN13548462, SAMN13548463, SAMN13548464, SAMN13548465, SAMN13548466, SAMN13548467, SAMN13548468, SAMN13548469, SAMN13548470, SAMN13548471, SAMN13548472, SAMN13548473, SAMN13548474, SAMN13548475, SAMN13548476, SAMN13548477, SAMN13548478, SAMN13548479, SAMN13548480, SAMN13548481, SAMN13548482, SAMN13548483, SAMN13548484, SAMN13548485, SAMN13548486, SAMN13548487, SAMN13548488, SAMN13548489, SAMN13548490, SAMN13548491, SAMN13548492, SAMN13548493, SAMN13548494, SAMN13548495, SAMN13548496, SAMN13548497, SAMN13548498, SAMN13548499, SAMN13548500, SAMN13548501, SAMN13548502, SAMN13548503, SAMN13548504, SAMN13548505, SAMN13548506, SAMN13548507, SAMN13548508, SAMN13548509, SAMN13548510, SAMN13548511, SAMN13548512, SAMN13548513, SAMN13548514, SAMN13548515, SAMN13548516, SAMN13548517, SAMN13548518, SAMN13548519, SAMN13548520, SAMN13548521, SAMN13548522, SAMN13548523.
