# Supplementary figures and images for: Evolutionary genomics of endangered Hawaiian tree snails (Achatinellidae: Achatinellinae) for conservation of adaptive capacity
Source: PeerJ. 2021 Apr 22;9:e10993. doi: 10.7717/peerj.10993 (PMC8071074; doi:10.7717/peerj.10993)

A

ML support

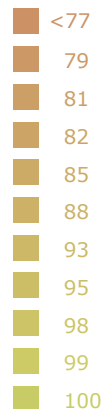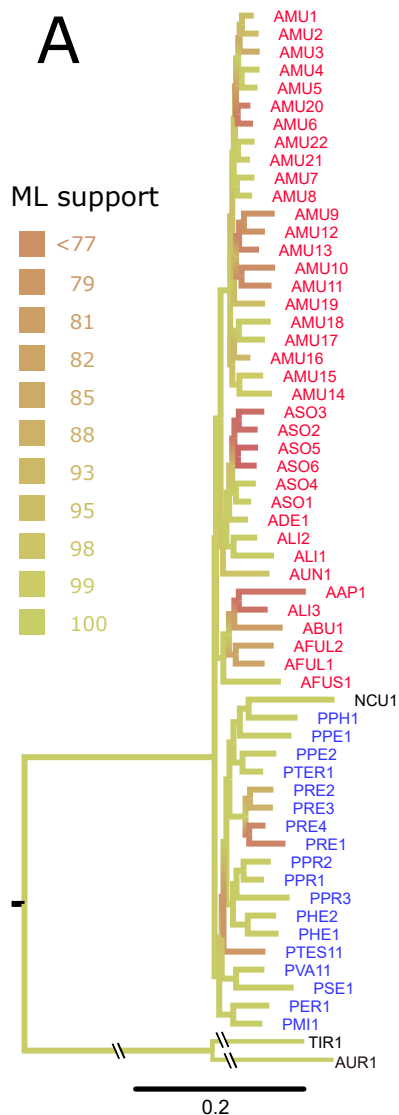

B

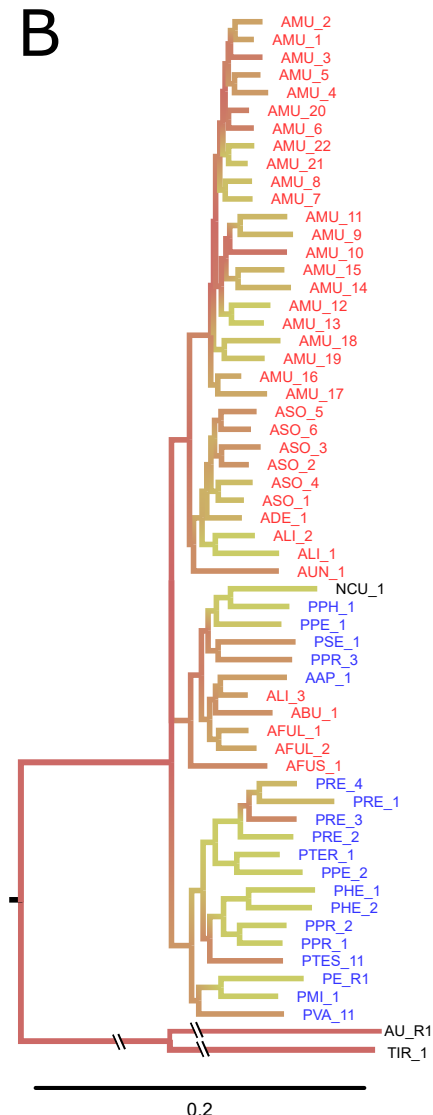

C

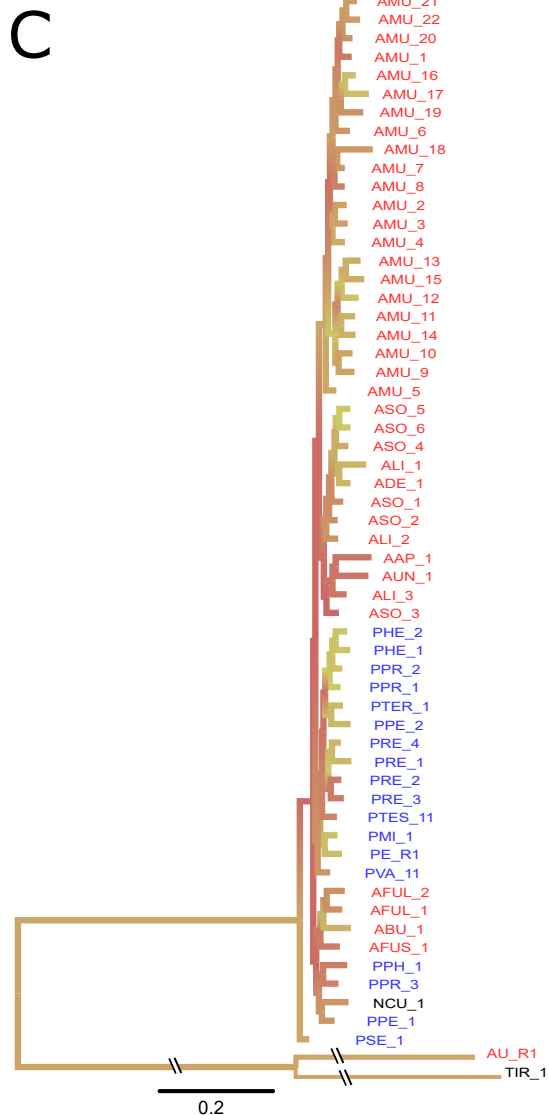

Supplement: Supplemental Information 1 — (A) ML tree of the ‘minimally filtered’ dataset that retained SNPs with a minimum quality score of 30, depth of 3x, with no limit on missing data per locus, resulting in 391,283 SNPs; (B) ML tree of the ‘medium filtered’ dataset retained SNPs with a minimum quality score of 30, depth of 5x, and a maximum of 5 missing taxa per locus resulting in 16,255 SNPs; (C) ML tree of the ‘strict filtered’ dataset retained SNPs with a minimum quality score of 40, depth of 10x, and a maximum of 10 missing taxa per locus resulting in 14,674 SNPs . Branches with ML (Maximum Likelihood) bootstrap support values above 90% are colored green, while values below ~77% are red, Achatinellidae are highlighted in red, while other genera are in blue font. The hash marks on the outgroup branches indicate that these branches are not to scale. [file peerj-09-10993-s001.pdf]
